# Structural Effects of Concrete Creep in a Prestressed Balanced Cantilever Bridge Based on Classical and Fractional Rheological Models

**DOI:** 10.3390/ma18235457

**Published:** 2025-12-03

**Authors:** Krzysztof Nowak, Radosław Oleszek, Artur Zbiciak

**Affiliations:** Faculty of Civil Engineering, Warsaw University of Technology, Al. Armii Ludowej 16, 00-637 Warsaw, Poland; radoslaw.oleszek@pw.edu.pl (R.O.); artur.zbiciak@pw.edu.pl (A.Z.)

**Keywords:** rheology, viscoelasticity, concrete creep, balanced cantilever method, prestressed bridge, fractional-order derivatives, fractional Zener model

## Abstract

This paper discusses the phenomenon of concrete creep and its impact on bridge structures, with particular emphasis on the mechanical models used to describe it. Classical rheological models, such as the Maxwell and Kelvin–Voigt, along with their generalized and fractional extensions incorporating fractional-order derivatives, are presented. These models differ in their complexity and in the accuracy of fit to laboratory test results. The use of non-classical, fractional-order rheological models (the fractional Kelvin–Voigt model and the fractional Zener model) enables better model fitting. The paper further describes methods for estimating creep effects in bridge design. The most popular is the effective modulus method, which is easy to implement but does not account for the load application history. More accurate approaches (e.g., Trost, Bažant, incremental method according to linear elasticity theory) are based on iterative procedures and require advanced computer implementation. The consequences of creep in bridge structures are highlighted: geometric (changes in elevation) and static (redistribution of internal forces and support reactions, changes in sectional stresses). These effects are particularly important in structures erected in stages, such as bridges built using the balanced cantilever method. The analytical section presents the influence of various creep models on changes in static quantities for a three-span prestressed bridge constructed by the cantilever method. The importance of proper selection of the creep model for the accuracy of engineering calculations and for the correct assessment of the long-term behavior of the structure is emphasized.

## 1. Introduction

The phenomenon of concrete creep under long-term loading is characterized by time-dependent strain accumulation, which can significantly affect the long-term behavior of structures [1]. One of the main consequences of bridge structures is deterioration of their functional properties, such as changes in the grade line, unacceptable increases in concrete stresses, or the development of tensile stresses that reduce the structure’s durability. In large-span bridges, underestimating creep effects can even lead to structural damage or failure [2]. Accurate modeling of creep is essential for predicting stress redistribution, displacement growth, and potential durability issues in reinforced and prestressed concrete elements. A variety of rheological models have been developed in the literature, ranging from classical mechanical systems to advanced models based on fractional calculus. Rheological models and their development have been comprehensively gathered and described in [3,4].

The most commonly used mechanical representations of creep behavior include the Maxwell and Kelvin–Voigt models, which simulate the material response using combinations of springs and dashpots [4]. Despite their computational advantages, these models often fail to accurately capture the full range of concrete creep behavior, particularly under complex loading conditions or over extended time periods.

To improve the accuracy of material behavior representation, more advanced rheological models have been introduced by combining multiple branches of Maxwell or Kelvin–Voigt elements. These generalized models offer better agreement with experimental data, but they require the identification of a large number of parameters, which complicates calibration and increases computational cost [4].

An alternative to classical models is provided by fractional-order rheological models, also known as fractional models [5]. In these models, classical dashpots are replaced with fractional elements. The inclusion of fractional-order elements in the rheological structure allows for good fitting of theoretical curves (creep, relaxation, hysteresis) to laboratory results with a significantly smaller number of rheological structure parameters to be determined through optimization (e.g., least squares method, genetic algorithm). This approach allows for better representation of material memory and hereditary effects while maintaining analytical tractability.

Recent studies have confirmed the effectiveness of fractional-order rheological models in describing concrete creep. The application of the fractional Kelvin–Voigt model to modeling concrete creep under constant load was presented in [6], where a calibration example is shown. In [7], various fractional models, including the fractional Kelvin–Voigt model, were used to describe the concrete creep under both constant and time-varying loads. In [8], the elastic-viscoelastic-plastic-viscoplastic creep model (MSSB, Modified Schofield-Scott-Blair model) was used to describe instantaneous creep and creep at a constant strain rate. To account for the nonlinear nature of concrete creep, the standard Newtonian dashpot in the MSSB model was replaced with a fractional element.

Beyond concrete structures, fractional-order models are used, for example, in the design of flexible and semi-rigid road pavements [9], as well as in the modeling of vibration isolation components in track structures, such as ballast [10] and ballastless [11], demonstrating their versatility.

This paper presents the application of fractional-order rheological models to the analysis of creep in prestressed concrete bridges constructed using the cantilever method. To date, fractional-order models have not been applied in bridge design practice. Thus, their implementation represents a significant novelty in bridge engineering, potentially transforming standard approaches to creep prediction.

The proposed methodology significantly improves the accuracy and reliability of time-dependent structural analyses. This enhancement is especially beneficial in cantilever bridge construction, where precise creep prediction is critical for ensuring long-term structural safety and performance.

## 2. Creep Phenomenon and Its Mechanical Models

**Figure 1:**
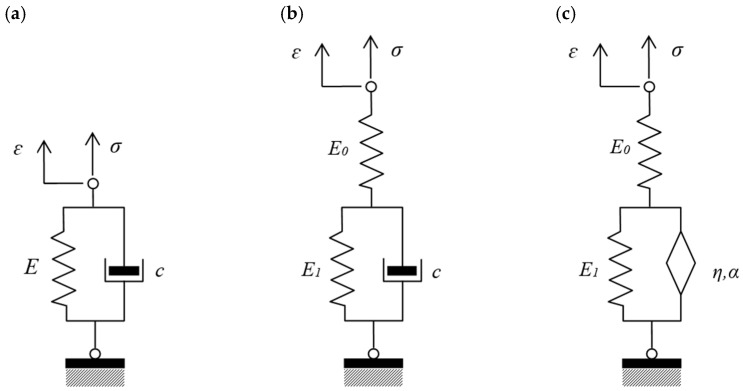
Mechanical models. E, $E_{0}$, and $E_{1}$ are elastic moduli of springs. c is the viscosity of the classical dashpot. η and α are the parameters of the fractional element. (**a**) Kelvin–Voigt model; (**b**) Zener model; (**c**) fractional Zener model.

The phenomenon of concrete creep under long-term constant loading is characterized by increasing deformations over time [1]. Various mechanical models are used to describe it, including two basic ones: Maxwell (series connection of a spring and a dashpot) and Kelvin–Voigt (parallel connection of a spring and a dashpot). The Kelvin–Voigt model is used in contemporary numerical models of building structures. It consists of two basic mechanical elements connected in parallel: an elastic element (Hookean spring) and a viscous element (Newtonian dashpot). The spring reflects the elastic properties of the material, including its ability to accumulate energy during deformation. The viscous element (dashpot) represents the phenomenon of energy dissipation. It is responsible, among other things, for delayed responses to changes in stress. The Kelvin–Voigt model is quite simple, which facilitates its numerical implementation. However, it does not always accurately reflect the real behavior of materials under complex loading conditions. Therefore, more advanced rheological models are used in engineering practice, depending on the specific material properties and operating conditions. For example, by connecting the Kelvin–Voigt model (Figure 1a) in series with an additional spring, we obtain the standard Zener model (Figure 1b). The fractional Zener model is obtained by replacing the viscous damper with a fractional element (Figure 1c).

Another method for constructing complex rheological structures that reflect the constitutive properties of viscoelastic materials involves appropriately combining multiple Maxwell or Kelvin–Voigt branches [12]. By connecting several Maxwell branches in parallel, we obtain the so-called Generalized Maxwell Model [12], which enables better fitting of material parameters to experimental results. A similar idea underlies the Generalized Kelvin–Voigt Model [12], which is constructed by connecting several Kelvin–Voigt branches in series. The use of generalized Maxwell or Kelvin–Voigt mechanical models requires determining numerous rheological parameters (spring and dashpot stiffness moduli) by fitting complex curve-fitting algorithms to experimental creep, relaxation, or hysteresis data.

**Figure 2:**
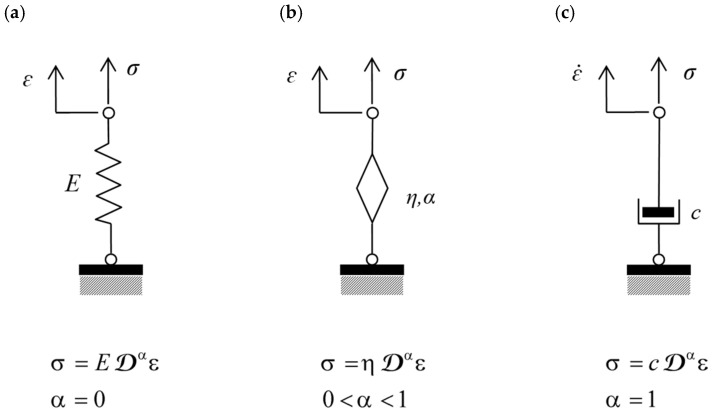
Elements of mechanical models: (**a**) Hookean spring with elastic modulus E. (**b**) Fractional element defined by parameters η and α. (**c**) Newtonian dashpot with viscosity c.

An alternative concept involves the use of so-called fractional-order rheological models (models described by fractional-order derivatives or fractional models) [13,14]. The fractional-order rheological element (fractional element) exhibits both the ability to accumulate and dissipate energy. Thus, it combines the concepts of a classical spring and dashpot (Figure 2).

In [15], the differential description of the fractional-order rheological structure, the so-called fractional Kelvin–Voigt model (FKV), is presented and analyzed, in which the viscous dashpot is replaced by a fractional element. The solution of the fractional differential equation describing the FKV model’s response to a unit stress step allows for the analytical form of the creep function. The solution uses the Mittag–Leffler function [16], and calculating its value at discrete times requires appropriate numerical techniques.

The fractional Kelvin–Voigt model is described by the following fractional-order differential equation of order α:

(1) $$\sigma \left(t\right)=E\varepsilon \left(t\right)+\eta \;\frac{d^{\alpha }\varepsilon \left(t\right)}{dt^{\alpha }},\;\;\;\;\;\;\alpha \in \left(0,1\right)$$

For a real number *α*, the Newton binomial has the following form:

(2) αk=α(α−1)⋯(α−k+1)k!=Γ(α+1)k!Γ(α−k+1)

where Γ denotes the gamma function.

The definition of the fractional-order derivative of order $\alpha \in R^{1}$ was given by Grünwald [16]:

(3) dαXtdtα=limh→0+1hα∑k=0N−1kαk·Xt−kh,    N=Entth

where Ent(z) determines the integer part of the real number z.

For α∈(0,1), we obtain the following:

(4) $$\frac{d^{\alpha }X\left(t\right)}{dt^{\alpha }}=\frac{X\left(0\right)}{\Gamma \left(1-\alpha \right)t^{\alpha }}+\frac{1}{\Gamma \left(1-\alpha \right)}\int_{0}^{t}\frac{\dot{X}\left(\tau \right)}{{\left(t-\tau \right)}^{\alpha }}d\tau$$

where

$$\Gamma \left(1-\alpha \right)=\int_{0}^{\infty }t^{-\alpha }e^{-t}dt$$

The solution of Equation (1) with respect to strains (creep test) has the following form:

(5) $$\varepsilon \left(t\right)=\frac{\sigma_{0}}{E}{\left(\frac{t}{\tau }\right)}^{\alpha }E_{\alpha ,\alpha +1}\left[-{\left(\frac{t}{\tau }\right)}^{\alpha }\right]\;$$

where

$$\tau ={\left(\frac{\eta }{E}\right)}^{1/\alpha };\eta =E\tau^{\alpha }$$

$E_{\alpha ,\beta }\left(z\right)≔\sum_{k=0}^{\infty }\frac{z^{k}}{\Gamma \left(\alpha k+\beta \right)}$ denotes the two-parameter Mittag–Leffler function.

It can be shown that for α→1 the fractional model reduces to the classical Kelvin–Voigt structure. This statement follows from the fact that for *α* → 1 the fractional derivative becomes the classical first derivative, and the Mittag–Leffler function tends to the exponential function. In this limit, the creep function of the fractional Kelvin–Voigt model reduces to the standard Kelvin–Voigt creep law, as discussed in [14,16].

In the case of the standard Zener model (Figure 1c), the creep function takes the form:

(6) $$J\left(t\right)=\frac{1}{E_{0}}+\frac{1}{E_{1}}{\left(\frac{t}{\tau }\right)}^{\alpha }E_{\alpha ,\alpha +1}\left[-{\left(\frac{t}{\tau }\right)}^{\alpha }\right]\;$$

Formula (6) can also be written as follows:

(7) $$J\left(t\right)=\frac{1}{E_{0}}+\frac{1}{E_{1}}\;\varphi (t,t_{0})$$

where

(8) $$\varphi \left(t,t_{0}\right)={\left(\frac{t}{\tau }\right)}^{\alpha }E_{\alpha ,\alpha +1}\left[-{\left(\frac{t}{\tau }\right)}^{\alpha }\right]$$

For $E_{0}=E_{1}=E_{c}$ Formula (7) reduces to the form of the creep function in the effective modulus method [17]:

(9) $$J\left(t\right)=\frac{1+\varphi (t,t_{0})}{E_{c}}$$

(10) $$E_{c,eff}=\frac{1}{J\left(t\right)}=\frac{E_{c}}{1+\varphi (t,t_{0})}$$

This analogy allows the use of the fractional Zener model (FZ Model) while retaining commonly used formulas. The use of non-classical, fractional rheological models enables better alignment between the mathematical model and experimental results [18].

## 3. Creep Effect in Bridge Structure Calculations

In bridge structures, concrete creep causes both geometric and static effects. Displacements and deformations of the structure during construction phases, induced by the introduction of self-weight and prestressing as successive cyclic segments are erected, as well as concrete creep, require the use of so-called construction camber (assembly camber) to achieve the designed elevation after the bridge is made continuous. After closure, the remaining concrete creep causes long-term changes in geometry. Regarding the static scheme, in bridges erected in stages, creep causes redistribution of internal forces and support reactions from self-weight and prestressing applied before continuity. In girder cross-sections, creep causes changes in stresses in concrete, reinforcing steel, and prestressing steel. All these effects can contribute to reduced crack resistance and even decompression of fully prestressed structures. The most perceptible effect for users is a change in design elevation during long-term operation. The scale of these phenomena depends primarily on the span and complexity of the structure.

To account for the influence of creep in bridge structure calculations, a range of simplified estimation methods has been developed over the years [15,19,20,21]. The most commonly used is the effective modulus method, in which creep is considered by correcting the concrete modulus of elasticity using the creep coefficient $\varphi (t,t_{0})$ [1,22]. This approach requires analyzing the structure in two separate numerical models: for short-term loads (with uncorrected modulus $E_{cm}$ and for long-term loads (with reduced modulus). The results are then summed. This method allows the use of widely available FEM systems, but its limitation is the inability to account for load application history. Moreover, it assumes complete reversibility of creep deformations, similar to the Kelvin–Voigt rheological model.

Another widely used approach, especially for simple structural systems built in stages (e.g., spans of precast beams), is the method of the corrected creep coefficient $C_{creep}$. Bridge structures made of precast beams are often designed as hyperstatic systems, in which precast elements are joined with reinforced concrete nodes. During assembly, the beams act as simply supported elements. Due to concrete creep under self-weight, there is a gradual increase in support moments in the composite structure, bringing the internal force system closer to that which would arise if the structure were built as continuous from the start. The age of the precast element at the time of incorporation significantly affects the value of the $C_{creep}$ coefficient.

More advanced approaches, such as the Trost method [23], the incremental method in the framework of linear elasticity theory, or Bažant models [2,24,25,26,27], require iterative algorithms and precise numerical implementation. Their use is mainly possible in specialized engineering FEM software such as SOFiSTiK, Midas, or Lusas [1,22,28]. These methods require creating an accurate numerical model, accounting for changes in the static scheme during subsequent construction and operation phases, as well as the full load and prestressing history of the structure. Knowledge of the rheological parameters of concrete is also crucial.

One of the first more precise methods accounting for creep was the so-called modified effective modulus method [23] (Trost, 1967). It was a development of the classical effective modulus method. In the Trost method, the sequence of applied loads is considered using a modified concrete modulus of elasticity:

(11) $$∆\varepsilon_{co}\left(t_{0}\right)+∆\varepsilon_{p}\left(t,t_{0}\right)=\frac{{\Delta \sigma }_{c}\left(t_{0}\right)}{E_{co,ef}}+\frac{{\Delta \sigma }_{c}\left(t\right)-{\Delta \sigma }_{c}\left(t_{0}\right)}{E_{co,m}}$$

where the modified modulus $E_{co,m}$ is calculated as follows:

(12) $$E_{co,m}=\frac{E_{c}(t_{0})}{1+\rho (t,t_{0})\varphi \left(t,t_{0}\right)}$$

$\rho \left(t,t_{0}\right)$—relaxation coefficient (Trost).

This method, implemented in SOFiSTiK, does not account for the effect of aging creep. In contrast, the Age-Adjusted Effective Modulus method (AAEM), proposed by Bažant in 1972 [24], allows for the consideration of aging creep. In AAEM, the classical modulus $E_{co,m}$ is replaced by $E^{\prime \prime }\left(t,t_{0}\right)$, and the relaxation coefficient by the aging coefficient $\chi \left(t,t_{0}\right)$. Both the effective modulus of elasticity $E^{\prime \prime }\left(t,t_{0}\right)$ and the aging coefficient $\chi \left(t,t_{0}\right)$ are time-dependent and depend on the age of the concrete at first loading ($t_{0}$).

(13) $$E^{\prime \prime }\left(t,t_{0}\right)=\frac{E\left(t_{0}\right)-R(t,t_{0})}{\phi (t,t_{0})}$$

where $R(t,t_{0})$ is the relaxation function. The aging coefficient can be determined by the following relationship:

(14) $$\chi \left(t,t_{0}\right)=\frac{E\left(t_{0}\right)}{E\left(t_{0}\right)-R(t,t_{0})}-\frac{1}{\phi (t,t_{0})}$$

Considering aging significantly increases the accuracy of calculations for loads applied at early concrete ages, which is important in technologies such as balanced cantilever construction or incremental launching.

The composition of the concrete mix (type of aggregate, admixtures, and additives) has a significant impact on the creep phenomenon, especially in high-strength concrete. In subsequent years, Bažant developed more complex creep and shrinkage models: model B3 [25,26,27] (Bažant, Baweja) and model B4 [28] (Bažant, Hubler, Wendner). Based on the analysis of the behavior of over 60 bridge structures worldwide [2], it was found that the slope of the deflection curve during the standard service period is consistently underestimated. Models B3 and B4 introduced a corrected course of the creep curve. Model B3 is based on calibration with short-term measurement results (1–3 months) and allows for an accurate reflection of the properties of specific concrete. Model parameters can be determined by linear regression. Further research developed a dataset from short-term laboratory tests, which was used to modify the equations in model B4, allowing the model to better reflect the long-term nature of creep.

An example of the calculation for Model B3 is presented in [26]. In Bažant models, creep under constant stress $\sigma_{c}\left(t\right)$, applied at time *t*_0_, is described as follows:

(15) $$\varepsilon_{c}\left(t\right)=\sigma_{c}\left(t\right)\cdot J\left(t,t_{0}\right)\;$$

where the creep function has the form:

(16) $$J\left(t,t_{0}\right)=q_{1}+\;C_{0}\left(t,t_{0}\right)+\;C_{d}\left(t,t_{0},t_{c}\right)$$

where

- $q_{1}=1/E_{0}$ is the inverse of the asymptotic elastic modulus, that is, the instantaneous deformation caused by unit stress,
- $C_{0}\left(t,t_{0}\right)$ is the compliance function for basic creep,
- $C_{d}\left(t,t_{0},t_{c}\right)$ is the compliance function accounting for drying creep,
- $t,t_{0},t_{c}$ respectively: the age of concrete at the considered time, the age of concrete at loading, and the time when drying started.

**Figure 3:**
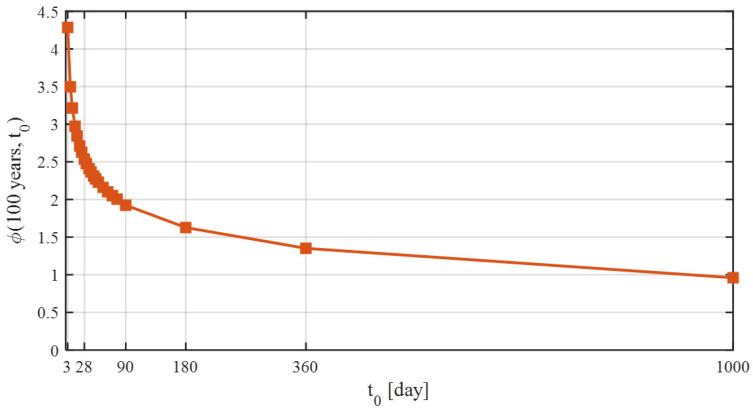
Value of the creep coefficient after 100 years, depending on the age of concrete at loading, according to the B3 model.

This approach assumes that creep can be decomposed into three components: aging viscoelastic, non-aging viscoelastic, and aging flow. An additional compliance function accounts for drying processes before first loading. The most important factors influencing creep include: the age of concrete at loading (Figure 3), concrete mix composition, cement type, ambient relative humidity, and effective thickness of the concrete element.

A particularly useful method for analyzing intermediate construction stages, whose properties vary along the structure (including bridges built using the balanced cantilever method), is the general incremental method. It is included in the EN 1992-2 standard [29]. It allows for consideration of both instantaneous deformations and increments of creep deformations in subsequent time intervals, depending on changes in stress. This approach enables analysis of the dependence of creep in a given section on the load application history.

The creep models included in EN 1992-1-1:2008 [17] assume an asymptotic limitation of creep curves, which is inconsistent with real observations indicating logarithmic long-term creep, as demonstrated in [2]. Models B3 and B4 reflect this phenomenon, assuming that the long-term asymptote of the creep curve is logarithmic. After several years of operation, both creep deformations and deflections increase linearly with respect to the logarithmic time scale.

The B3 model was used to calibrate the fractional Zener model. The paper presents the influence of two different creep models (according to EN and the fractional Zener model) on the static quantities of a prestressed concrete bridge constructed by the balanced cantilever method.

## 4. Influence of the Creep Model on Bridge Statics

### 4.1. Description of the Analyzed Structure

**Figure 4:**
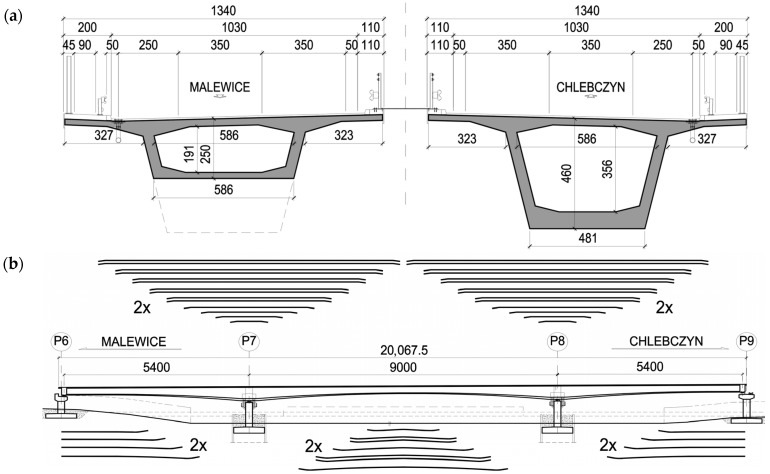
Geometric and structural solutions of the analyzed bridge (dimensions in cm): (**a**) cross-section of span and support, (**b**) longitudinal section with the layout of prestressing tendons.

The bridge considered in the paper is a three-span continuous beam with spans of 54.0 + 90.0 + 54.0 m, constructed using the balanced cantilever method (Figure 4). The total length is 200.68 m, the width of each carriageway is 13.40 m, and the crossing angle is 90°. The superstructure will be supported on monolithic wall piers founded on piles.

**Figure 5:**
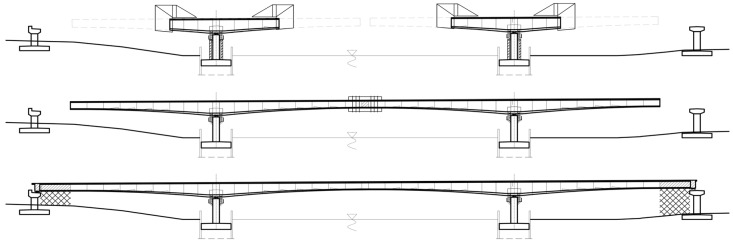
Construction technology of the designed bridge—cantilever casting method.

The structure is designed for load class in accordance with EN standards [17,29,30,31,32,33,34,35], considering national adjustment factors for class I live load. The load-bearing girder is a single-cell box section made of C50/60 concrete (Figure 4a), prestressed with 19-strand cables of steel class $f_{pk}=1860\;MPa$ reinforced with high-ductility steel $f_{yk}=500\;MPa$. In the upper slab above the supports, 28 L19 cables are provided; in the lower slab of the main span, 14 L19 tendons; and the end spans will be prestressed with 8 L19 cables located in the lower slab (Figure 4b). The superstructure is designed using mixed technology. The main span and its closure segment, as well as about 4/5 of the end spans, will be constructed using the balanced cantilever method, while the remaining parts of these spans (about 1/5 of the span length) will be built conventionally on stationary scaffolding (Figure 5).

Construction using the cantilever method will begin with the execution of the starter segment SM-1 at the intermediate supports, on traditional scaffolding supported on the foundation. Mobile form travelers will be installed on this part of the structure, moving along the prestressed starter segment. It will be supported during assembly by concrete columns prestressed to the bottom slab of the box girder. In subsequent phases, the next segments will be constructed on both sides of the cantilever. In the final stage, the closure segment (concreting and prestressing) and the monolithic parts of the end spans will be executed.

### 4.2. Assumptions and FEM Model

The EN standards [17,29,30,31,32,33,34,35] do not specify as clear rules for the strength design of prestressed concrete bridges as the withdrawn regulations, based on which most balanced cantilever bridges in Poland were built. Therefore, based on Eurocode requirements and the authors’ experience and technical literature [36,37,38,39,40], a series of detailed design assumptions were adopted.

During the balanced cantilever construction stages, full prestressing was assumed, i.e., no tensile stresses occur in the deck slab in the vicinity of prestressing cables. In the service phase, after closure, under the characteristic SLS combination, limited prestressing is assumed, i.e., $\sigma_{ct}<f_{ctk0.05}=2.90\;MPa$. In the service stage, for the frequent load combination in SLS (including about 60–70% of live loads UDL + TS), the decompression condition according to EN [29] is ensured, i.e., the prestressing tendons are covered with 10 cm of compressed concrete, which corresponds to full prestressing (no tension). For all load combinations relevant to the serviceability limit states (SLS), cracking of the span is not allowed (i.e., no flexural cracks). The maximum compressive stresses in concrete for the characteristic SLS combination in the assembly and service stages satisfy the condition $\sigma_{c}\leq 0.6\;f_{ck}=30.0\;MPa$. The ultimate bending capacity of critical sections in the ULS design combination was determined considering bonded prestressing tendons (with full adhesion) according to 6.1(2) EN 1992-1-1 [17]. The amount of conventional reinforcement results from the ultimate capacity condition according to 6.1 EN 1992-1-1 and EN 1992-2 [17,29] and protection against brittle failure according to 5.10.1(106) and to 6.1(109) EN 1992-2 [29].

**Figure 6:**
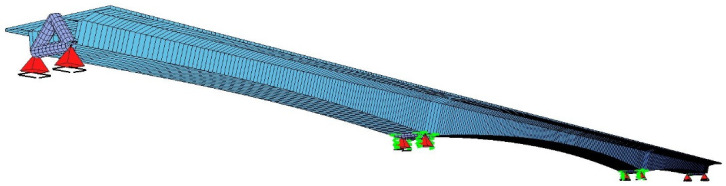
Numerical model of the bridge developed in the SOFiSTiK FEM environment—spatial beam model for global analysis of span behavior and erection stages (e1, p3).

For the design of the structure, a beam model in three-dimensional space (class e1, p3) was developed in the SOFiSTiK FEM environment, which, due to the eccentricity of the bearings, can be described as a spatial frame (Figure 6). The maximum length of the beam element is 0.5 m. The concrete material model was generated automatically in SOFiSTiK.

**Figure 7:**
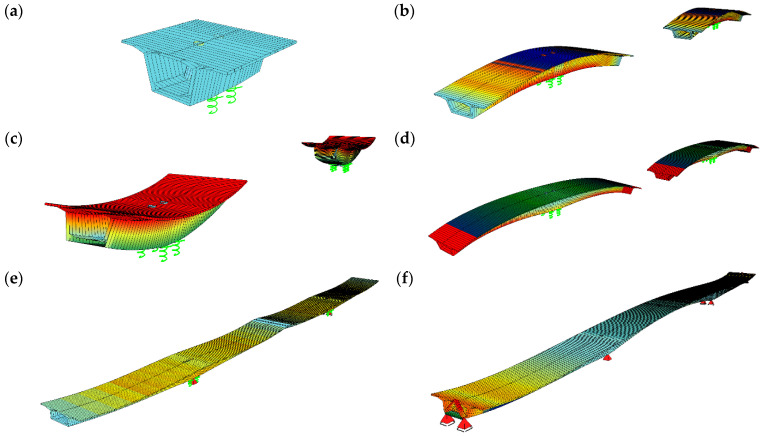
Example analysis of the bridge construction sequence: (**a**) initial segment SM, (**b**) stage SM-5, (**c**) stage SM-3—segment prestressing, (**d**) stage SM-7—effect of traveler weight, (**e**) stage SM-10—closure segment, (**f**) stage SM-11—monolithic segments. Support notation: Temporary (green springs); Final bearings (red pyramids).

This model allowed for the simulation of the balanced cantilever construction scenario (changes in the static scheme and load history), rheological phenomena, selection of prestressing cable layouts, and strength design of the span (verification of SLS stresses, ultimate capacity in ULS, and design of conventional reinforcement). The structure was analyzed from the first construction stages to the final static scheme, accounting for redistribution of internal forces and stresses due to creep using a single model (Figure 7).

The calculations included structure loads according to EN [17,29,30,31,32,33,34,35], considering national adjustment factors for class I live load. Additionally, during the balanced cantilever construction phases, technological and assembly effects [36,39,40,41,42,43], were included, such as self-weight with allowance for execution inaccuracies, concrete shrinkage and creep during construction progress, prestressing effects, distributed and concentrated technological loads (equipment and tools, workers), traveler weights, wind pressure, cantilever alignment before closure, closure segment formwork, etc. The characteristic, frequent, and quasi-permanent combinations in SLS and the permanent and exceptional combinations in ULS were considered.

The bridge FEM model reflected intermediate assembly states (construction phases) using the CSM (Construction Stage Manager) module for modeling cycles and construction scenarios in SOFiSTiK. Changes in the static scheme, load system, and rheological effects (creep and shrinkage) on the state of stresses and displacements of the spans were considered. The CSM module enables automated determination of creep and shrinkage during construction progress, which is crucial for balanced cantilever construction. The system uses the incremental (recursive) Trost method and relationships from EN [17,29,30,31,32,33,34,35]. It also allows for the introduction of custom creep curves.

### 4.3. Creep Models Used in Bridge Design

To assess the influence of the concrete creep model on the distribution of static quantities in the structure, four computational simulations of the bridge (W1, W2, W3, W4) were carried out using the same geometric model. In cases W1 and W2, prestressing of successive segments was assumed after 3 days from concreting (7-day cycle), while in W3 and W4, after 7 days (10-day cycle). In analyses W1 and W3, standard EN relationships [17,29] and the incremental Trost-Bažant method (modified effective modulus method) available in the system were used. In calculations W2 and W4, a more advanced fractional Zener creep model was implemented. The fractional Zener model was calibrated based on the B3 model [25,26,27] accounting for the C50/60 concrete mix composition, i.e., cement (465 kg/m^3^) and aggregate content (1930 kg/m^3^), w/c ratio (0.41), cement type (high early strength), age at first loading, relative humidity (0.80), and the volume-to-surface ratio of the concrete element (12 cm).

**Figure 8:**
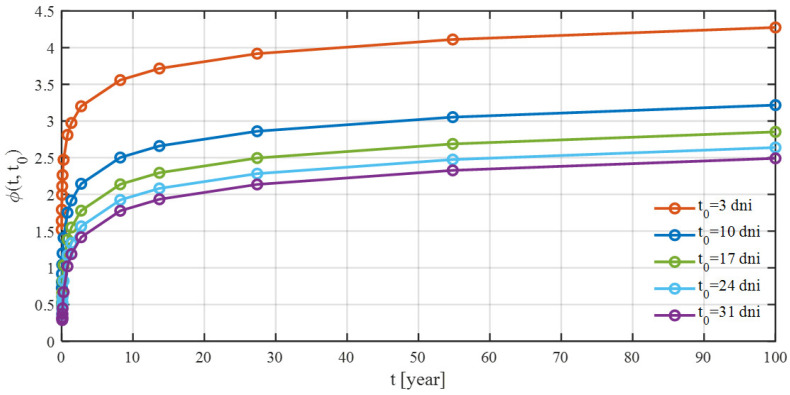
Graph of creep coefficient curves according to the B3 model for various loading ages of concrete.

To accurately capture creep, a total of 22 curves describing its progression over time were generated using the B3 model. Each of these curves corresponds to the considered age of concrete at loading (3, 7, 10, 14, 17, 21, 24, 28, 31, 35, 38, 42, 45, 49, 56, 63, 70, 77, 90, 180, 360, and 1000 days). For bridges constructed using the balanced cantilever method, the age of concrete at loading varies between segments cast in successive construction cycles. For instance, assuming a 7-day construction cycle with prestressing applied 3 days after concreting, when prestressing segment SM-3, the concrete age at loading is 3 days. In the previously completed segments, SM-2 and SM-1, the concrete ages are 10 and 17 days, respectively. The change in the creep curve in the short term after concreting is very significant (Figure 8).

It should be noted that the curves shown in Figure 8 can also be obtained independently using the fractional models described in Section 2. Unlike the B3 model, the applied fractional Zener model is described by differential equations. As a result, it allows not only for the generation of creep curves but also for the simulation of the material’s response to arbitrary loading conditions. The model enables the analysis of creep, relaxation, and cyclic loading, which will be further developed and elaborated upon in the authors’ forthcoming papers.

The fractional Zener model was calibrated to curves generated by the B3 model for different ages of concrete at loading. The FZ model includes four parameters (Figure 1c). For $E_{0}=E_{1}=E$ the number of parameters requiring calibration is reduced to three: $E\;\left[Pa\right]$, $\eta \;[Pa\cdot s^{\alpha }]$ and α [−]. Instead of η, the parameter τ [s], can also be used as shown in Equation (5). The exact calibration procedure for fractional models is described in [44]. The calibration procedure consists of determining the optimal parameter values $p_{opt}\in R^{m}$, where m denotes the number of parameters. The most commonly used method for model fitting to data is the least squares method, which minimizes the error between the experimentally determined values of the creep coefficient $\varphi_{exp}(t_{i})$ for successive time points $t_{i}$ and the values predicted by the model $\varphi_{mod}(t_{i},p)$.

(17) $$p_{opt}={\arg\underset{p\in \Omega }{\min}\sum_{i}\left[\varphi_{exp}\left(t_{i}\right)-\varphi_{mod}\left(t_{i},p\right)\right]}^{2}$$

where Ω is the admissible parameter space, bounded by lower $p_{L}\in R^{m}$ and upper $p_{U}\in R^{m}$ parameter limits:

(18) $$\Omega ∶=\left\{p\in R^{m}:\;p_{L}\leq p\leq p_{U}\right\}$$

In practice, computational tools such as the *lsqcurvefit* function available in MATLAB R2024b are used to solve such problems, implementing efficient numerical algorithms for fast and accurate identification of optimal parameters. The solver iteratively modifies parameter values until the best model fit to actual data is achieved.

For calibration of the fractional Zener model, a proprietary calibration spreadsheet was prepared in MATLAB R2024b, into which data comprising the values of the creep coefficient determined according to the B3 model and the time points at which these values were determined were imported. The algorithm implemented the equation for the creep function (9), where the creep coefficient is determined according to (8).

**Figure 9:**
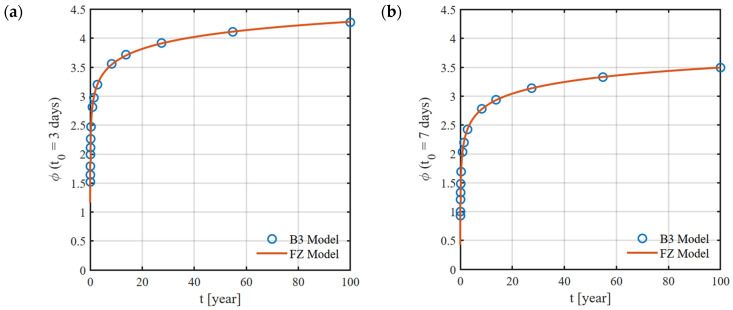
Creep coefficient plots obtained for the FZ model: (**a**) $t_{0}$ = 3 days, (**b**) $t_{0}$ = 7 days.

After calibration, for each curve generated by the B3 model, a set of optimal parameters (E,η, α) was obtained, by which the creep function and the creep coefficient function of the fractional model are described. The optimal parameter values for different ages of concrete at loading $t_{0}$ are presented in Table 1. Sample creep coefficient curves for the age of concrete at loading ($t_{0}$ = 3 days and 7 days) are shown in Figure 9.

**Figure 10:**
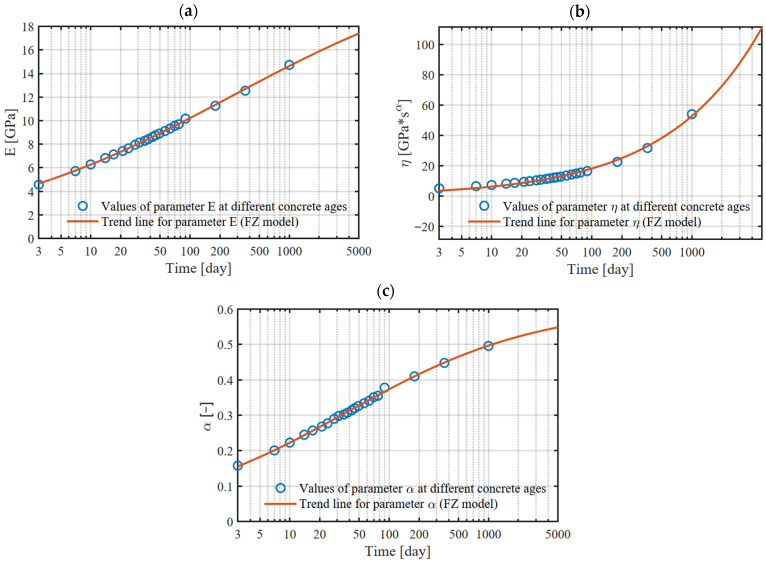
Values of parameters in the FZ model for concrete loaded at different ages: (**a**) E[GPa], (**b**) $\eta [GPa\cdot {year}^{\alpha }]$, (**c**) α[−].

For ages of concrete at loading not defined by a creep curve, the SOFiSTiK software does not interpolate but automatically applies the function for the nearest defined age. Therefore, in calculations W2 and W4, many creep curves corresponding to the loading of early-age concrete were used. Each of these curves was described by the fractional Zener model. The values of the parameters describing these curves are presented in Table 1. When using individual creep curves defined in SOFiSTiK, a compatible shrinkage curve must also be specified. Due to the lesser significance of the age of concrete at loading on shrinkage, a single curve was defined to describe this phenomenon. The obtained parameter sets are presented in the graphs as a function of the concrete age at the time of first loading (Figure 10). The trend lines for the parameters of the fractional Zener model were fitted using the Mittag–Leffler function, which provides an accurate representation of parameter evolution during concrete maturation.

The calibration of the fractional Zener model presented in this study is based on numerical data generated by the B3 model. Direct laboratory validation of the calibrated parameters is planned in future work to further confirm the accuracy of the proposed approach.

The parameters *E*, *η*, *α*, and *τ* describe the elastic stiffness, viscous effects, fractional material memory, and characteristic time of the viscoelastic response. A full sensitivity study of these parameters would give deeper insight into their individual influence on creep. The present work is already extensive and covers both model calibration and a complete structural analysis of a balanced cantilever bridge. For this reason, a detailed sensitivity analysis is planned for future work as a separate study.

### 4.4. Internal Forces and Support Reactions

The creep phenomenon in prestressed concrete structures is induced by prestressing, self-weight, and other long-term actions. To thoroughly investigate its effects from individual loads in each analyzed case (W1–W4), two variants were considered: A, with long-term actions without prestressing; B, with prestressing as the only load.

For long-term loads (except prestressing), during the assembly phases (cantilever construction), loading successive segments does not change internal forces (isostatic system). The effect of concrete creep from long-term loads becomes apparent from the moment of structure closure and persists for the long term (100-year period). As a result of concrete creep, from the moment of structure continuity, the distribution of internal forces due to self-weight tends toward the state that would arise if the structure were built as continuous from the beginning. The bridge begins to operate as a hyperstatic system after the introduction of most permanent loads (causing creep). The effect of almost the entire self-weight is considered in isostatic (cantilever) schemes. Only after the removal of the monolithic end-span sections (10 m) built on stationary scaffolding do their weight begin to act on the three-span continuous beam.

**Figure 11:**
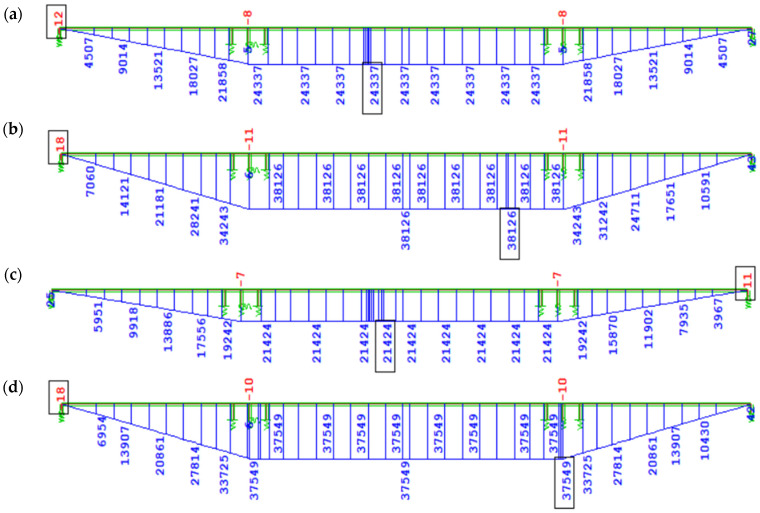
Bending moment increase ∆My [kNm] due to creep after 100 years of service since structural closure from long-term loading without prestressing: (**a**) W1, EN 3 days; (**b**) W2, FZ 3 days; (**c**) W3, EN 7 days; (**d**) W4, FZ 7 days.

In cases W1 and W3, based on the Eurocode creep model, the increments of bending moment ∆My, induced by creep from long-term loads without prestressing after 100 years of operation, are similar, with a difference of 13.6%—for W1, the increment ∆My = 24,337 kNm, while for W3—∆My = 21,424 kNm (Figure 11a,c). The discrepancies in ∆My values in variants W2 and W4 (Figure 11b,d) according to the FZ creep model are negligible (1.5%).

A significant difference in ∆My values of about 17,000 kNm, induced by creep from long-term loads (mainly self-weight), was observed when comparing the EN description (W1 and W3) to the FZ model (W2 and W4) (see Figure 11c,d). This results from the underestimation of the creep coefficient φ(t) by Eurocode procedures for concrete loaded at an early age, compared to the FZ model. It should be noted that in the Trost method, failure to account for aging during loading leads to underestimation of creep effects for concrete loaded at an early age.

**Figure 12:**
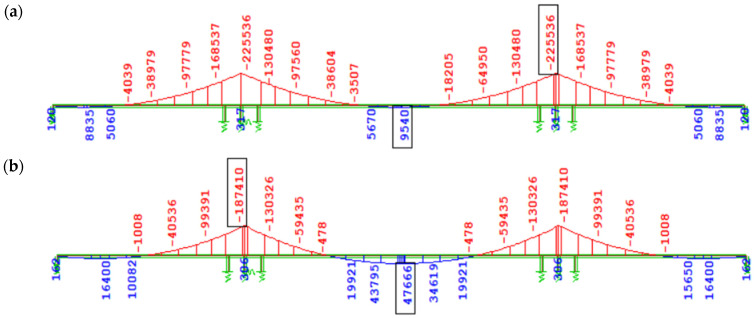
Bending moment My [kNm] for analysis 2 (FZ model, 3 days): (**a**) from self-weight just after completion, (**b**) from self-weight plus creep after 100 years.

Figure 12 shows the bending moment diagram My due to self-weight immediately after full continuity of the structure (Figure 12a), as well as its change accounting for 100-year concrete creep (Figure 12b) in variant W2 with the FZ model (prestressing applied on day 3). In the remaining cases, the nature of these diagrams is similar.

Prestressing the structure causes long-term creep (from the moment of structure closure) and, during the cantilever phases, already causes a slight reduction in prestressing force (during construction, part of its rheological losses occur). It is worth noting that most of the prestressing force (84%) is introduced into the structure through the isostatic system (two cantilevers or a single-span beam with cantilevers), with only about 15% in the final monolithic end spans. After prestressing, the system begins to function continuously, and only from that moment, substitute forces induced by creep arise in the superstructure, resulting from the staged construction technology.

**Figure 13:**
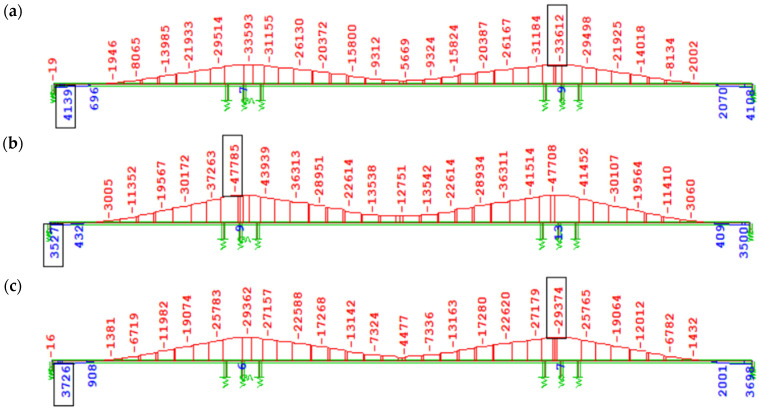

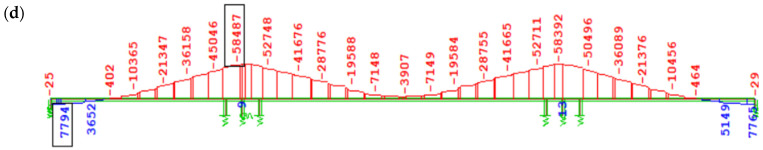
Bending moment increase ∆My [kNm] due to creep after 100 years of service since structural closure induced solely by prestressing: (**a**) W1, EN 3 days, (**b**) W2, FZ 3 days, (**c**) W3, EN 7 days, (**d**) W4, FZ 7 days.

Figure 13 presents the increase in bending moment ∆M_y_ due to creep induced solely by the prestressing of the structure, accounting for long-term creep effects (excluding creep during the cantilever stages, which has a minor impact on the overall internal forces due to the reduction in prestressing force). In the case of prestressing effects in variants using the creep model from EN, similarly to the self-weight load, no significant differences in bending moment values were observed depending on the concrete age at the time of prestressing of subsequent segments (3 days, Figure 13a; 7 days, Figure 13c). In variant W2 (Figure 13b), according to the FZ model and prestressing of successive segments at 3 days, a much greater absolute increment of negative bending moment (tensile in the upper fibers) from creep was recorded compared to W1 (Figure 13a), i.e., |∆My| ≈ 14,000 kNm over the support and |∆My| ≈ 7000 kNm in the span. In variant W4 (Figure 13d), according to the FZ model and prestressing at 7 days compared to W3 (Figure 13c) according to EN, the increment of bending moment over the support from creep induced only by prestressing increased almost twofold (by about 29,000 kNm).

**Figure 14:**
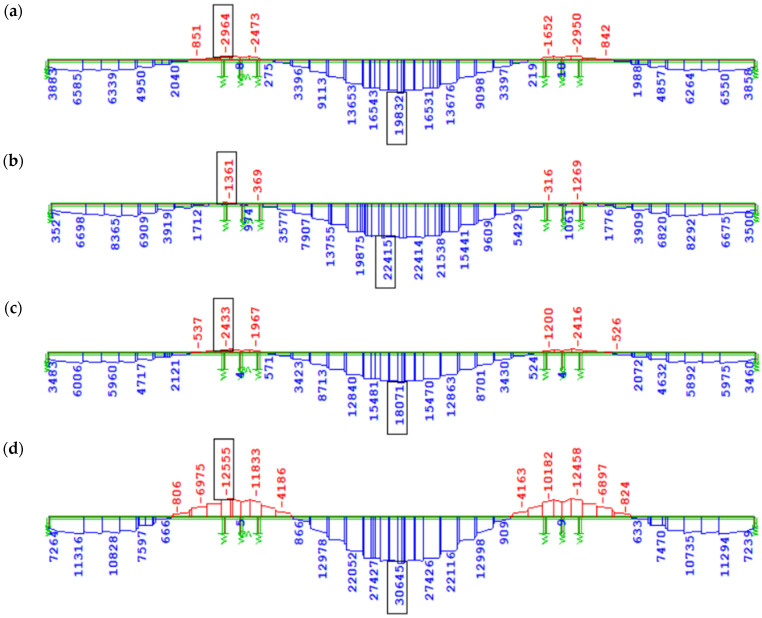
Bending moment changes ∆My [kNm] due to creep after 100 years of service since structural closure from all long-term loads: (**a**) W1, EN 3 days; (**b**) W2, FZ 3 days; (**c**) W3, EN 7 days; (**d**) W4, FZ 7 days.

Figure 14 shows the increase in the bending moment ∆My caused by the creep of concrete from all long-term loads after 100 years of the structure’s service life, counted from the closure of the structure.

No significant influence of the creep model on support reactions was observed in the analyses, with differences at intermediate supports below 1%. At end supports, due to smaller loads, the discrepancies in uplift reactions reach 5% (W1—764 kN, W2—724 kN), while the differences in maximum reactions oscillate around 1%.

### 4.5. Stress State of the Bridge Structure

**Figure 15:**
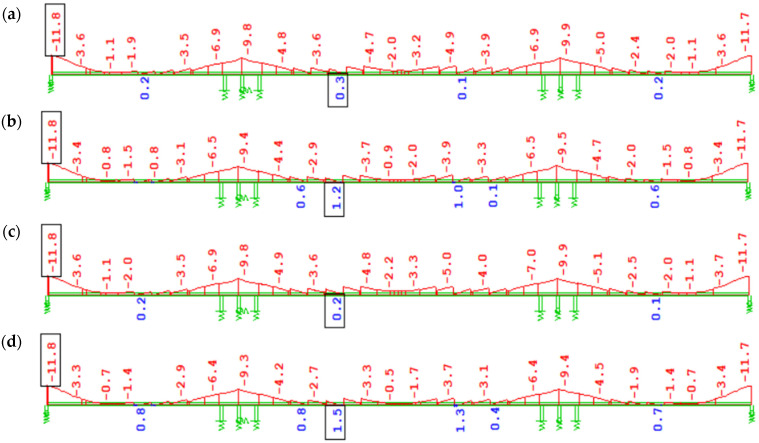
Stresses in bottom fibers under characteristic SLS combination from all loads [MPa]: (**a**) W1, EN 3 days; (**b**) W2, FZ 3 days; (**c**) W3, EN 7 days; (**d**) W4, FZ 7 days.

An important aspect of assessing the influence of the concrete creep model on bridge statics is the comparison of the stress state in the analyzed variants. Its undesirable effect may be an unacceptable increase in compressive stresses in concrete or the appearance of tensile stresses, both of which reduce crack resistance and durability. In the no-load combination, with respect to serviceability limit states (SLS), cracking of the span is allowed (i.e., no flexural cracks). The influence of the creep model on the stress state is shown in Figure 15.

According to the design assumptions (Section 4.2), during the balanced cantilever construction stages, full prestressing (no tension) was assumed, i.e., no tensile stresses occur in the deck slab near the prestressing cables. In the service phase after structure closure, under the characteristic SLS combination, limited prestressing (no cracking) was assumed, i.e., $\sigma_{ct}<f_{ctk0.05}=2.90\;MPa$. The use of a more advanced concrete creep model, compared to the normative EN proposals, allows for more precise estimation of creep effects, including the increase in tensile stresses, which is important for assessing crack resistance. The difference in tensile stresses in the bottom fibers of the girder in the characteristic combination is 0.9–1.3 MPa (Figure 15). Their values obtained in analyses with the FZ model (W2—1.2 MPa, W4—1.5 MPa) do not exceed the concrete tensile strength $f_{ctk0.05}=2.90\;MPa$. Extending the duration of assembly cycles and delaying the prestressing of successive segments negatively affects the expected values of tensile stresses in the final structure. In variant W4 (prestressing after 7 days), tensile stresses were 0.3 MPa higher than in W2 (prestressing after 3 days). Such small differences, however, are within the accuracy of engineering calculations.

### 4.6. Structural Displacements

**Figure 16:**
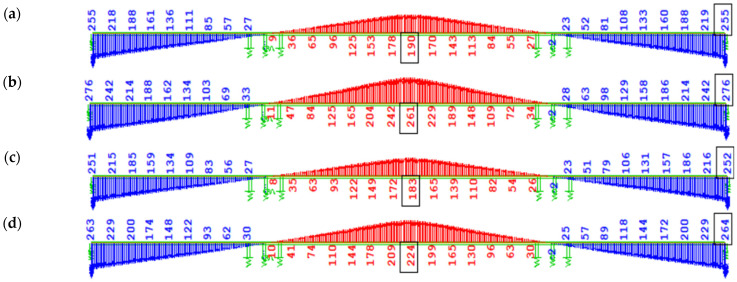
Displacements due to long-term loads immediately after the completion [mm]: (**a**) W1, EN 3 days; (**b**) W2, FZ 3 days; (**c**) W3, EN 7 days; (**d**) W4, FZ 7 days.

**Figure 17:**
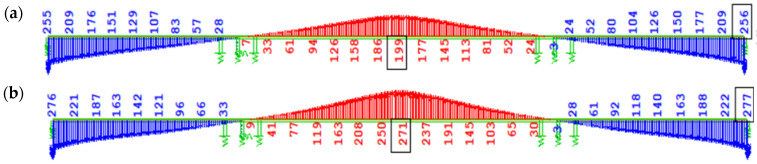
Displacements of the structure due to long-term loads, including creep after 100 years of service [mm]: (**a**) W1, EN 3 days; (**b**) W2, FZ 3 days.

Concrete creep causing excessive structural deformations and changes in the designed elevation may lead to deterioration of the bridge’s service properties. In this respect, precise prediction of vertical displacements of the superstructure during individual construction stages is crucial. This has a fundamental impact on the selection of so-called assembly cambers for individual bridge segments to achieve the designed elevation after closure and continuity of the structure. Incorrect estimation of superstructure displacements during assembly (cantilever phases) may necessitate introducing a higher-than-assumed force to align the cantilevers. The influence of the creep model on displacements before full continuity and completion of the final monolithic segments is illustrated in Figure 16 and Figure 17.

The differences in displacements estimated by analyses with the EN and FZ creep models, immediately after completion of the structure, are shown in Figure 16. In the case of prestressing after 3 days from segment concreting (7-day cycle), the difference is 71 mm. The displacement obtained in variant W2 with the FZ model is 261 mm (Figure 16b), while in analysis W1 according to EN, it is 190 mm (Figure 16a). For prestressing after 7 days from segment concreting (10-day cycle), the displacement difference is 41 mm. In variant W4 (FZ model), the total displacement was estimated at 224 mm (Figure 16d), while in W3, according to EN, it is 183 mm (Figure 16c).

The duration of assembly cycles and the age of concrete at prestressing affect vertical displacements. It is important to adhere to the technological regimes assumed at the design stage throughout the construction process.

The vertical displacement state of the structure from the start of the, partially offset each other. The nature of displacement change (downward or upward) depends on the dominant influence of one of these effects and the distribution of compressive stresses along the height of the box girder. In the analyzed structure, the long-term predicted change in displacement due to concrete creep is small (upward deflection of 9–10 mm) during operation (see Figure 16a,b and Figure 17a,b). The bridge’s service life may change due to long-term rheological processes (concrete shrinkage and creep, prestressing steel relaxation). The effects of creep from self-weight and prestressing of the superstructure, including displacements.

## 5. Main Results

Table 2 presents the key results in the critical sections obtained from the different computational simulations.

The differences in tensile stresses in the bottom fibers of the box girder are 0.9–1.3 MPa (characteristic combination), and their values obtained in the FZ model (1.2–1.5 MPa) do not exceed the concrete tensile strength $f_{ctk0.05}=2.90\;MPa$.

The differences in estimated vertical displacements of the main span immediately after structure closure reach 71 mm for prestressing after 3 days from segment concreting (7-day cycle) and 41 mm for prestressing after 7 days (10-day cycle).

## 6. Conclusions

The paper illustrates the influence of different concrete creep models on the values and distribution of static quantities in a bridge structure constructed by the balanced cantilever method.

After analyzing the variants using the concrete creep model according to EN and the fractional Zener model calibrated on the B3, it should be stated that the Eurocode approach leads to an underestimation of the effects of this phenomenon. Creep occurring during the cantilever construction phase does not cause changes (redistribution) of internal forces in the isostatic system (only indirectly causing a slight reduction in prestressing force). The values of substitute forces induced by creep become clearly visible only during long-term (100-year) creep. The age of concrete at loading and the duration of assembly cycles determine which part of the total creep occurs during the cantilever phase and what part will still occur in the final (continuous) static scheme.

The use of advanced concrete creep models rather than the normative EN proposals allows for more precise estimation of creep effects, including the increase in tensile stresses, which is important for assessing crack resistance. Extending cycle duration and delaying prestressing of successive segments negatively affect the stress state of the final structure. A rise in tensile stresses in the bottom fibers of the span zones was observed.

The influence of the adopted concrete creep model is visible when assessing the history (sequence) of changes in vertical displacements during the construction phases by the balanced cantilever method. The use of more mathematically and physically advanced creep models enables more precise prediction of superstructure displacements at individual construction stages. This is crucial for selecting the so-called assembly cambers for individual bridge segments to achieve the designed elevation after closure and continuity. It should be remembered that the values of deflections during the cantilever phase depend on the duration of assembly cycles and the age of concrete at prestressing. In this respect, it is crucial to maintain the technological regimes throughout the construction process, as assumed at the design stage.

For the analyzed bridge structure, the influence of the concrete creep model on the scale of redistribution of internal forces and the final stress state during long-term operation from the moment of structure closure should be considered minor. The adopted concrete creep model is also of lesser importance in terms of the ultimate capacity of the structure. The accuracy of predicting the range of redistribution of internal forces from self-weight has no practical significance in this case, as this load causes only part of the total internal forces induced by other loads. In summary, for the class of balanced cantilever bridges with a relatively small main span (90.0 m), the influence of the concrete creep model is crucial only when determining the so-called assembly cambers for individual bridge segments. Underestimating the creep effect during construction may result in deviations from the designed deck elevation after completion (the deck in midspan being lower than intended), which has operational significance. For this class of balanced cantilever bridges, the use of the approach proposed in EN has no major structural importance. It is to be expected that for structures with larger spans, the influence of the creep model on the scale of internal force redistribution, the final stress state, and the values of displacements during long-term service will be greater.

## 7. Scope and Novelty of the Present Study

The study includes model formulation, calibration, implementation in a commercial FEM system, and detailed bridge design stages.

The main novelties of this study are as follows:

- Implementation of fractional rheological models in bridge engineering for more accurate creep prediction with fewer parameters.
- Development of calibrated parameter sets for the fractional Zener model, tailored to different concrete loading ages, based on B3 model data.
- Implementation of fractional Zener Creep Curves in SOFiSTiK—Integration of 22 creep curves reflecting various segment ages and construction stages.

These advancements improve the accuracy of time-dependent structural analysis and are particularly valuable in the staged construction of balanced cantilever bridges.

To support the numerical comparison, it is important to note that the long-term creep trend predicted by the fractional Zener model agrees with experimental observations. Laboratory tests on concrete creep presented in [6,7], and long-term monitoring of concrete structures discussed in [2] show a logarithmic increase in creep strains over time. The Eurocode model does not reproduce this behavior. The fractional Zener model calibrated on the B3 curves follows the experimental trend with higher accuracy. This confirms that the use of fractional models is supported not only by numerical results but also by existing experimental evidence.

## Figures and Tables

**Table 1:** Parameters of the Zener model and the creep coefficient value after 100 years.

$t_{0}\;[day]$	3	7	10	14	17	21	24	28	31	35	38
E [GPa]	4.57	5.72	6.28	6.82	7.13	7.42	7.65	7.95	8.14	8.29	8.43
$\eta \;[GPa\cdot {year}^{\alpha }]$	5.16	6.60	7.37	8.21	8.77	9.47	9.92	10.46	10.85	11.37	11.73
α [−]	0.158	0.201	0.223	0.245	0.257	0.268	0.277	0.290	0.298	0.302	0.307
τ [year]	2.16	2.04	2.06	2.14	2.24	2.48	2.56	2.57	2.62	2.85	2.93
$\varphi (100\;years,t_{0})$	4.29	3.50	3.21	2.97	2.84	2.71	2.63	2.54	2.48	2.41	2.37
$t_{0}\;[day]$	42	45	49	56	63	70	77	90	180	360	1000
E [GPa]	8.62	8.76	8.90	9.12	9.33	9.55	9.69	10.17	11.26	12.54	14.73
$\eta \;[GPa\cdot {year}^{\alpha }]$	12.16	12.47	12.88	13.63	14.28	14.90	15.51	16.55	22.60	31.72	54.02
α [−]	0.314	0.320	0.326	0.334	0.341	0.351	0.355	0.378	0.410	0.448	0.496
τ [year]	2.99	3.01	3.11	3.32	3.48	3.55	3.77	3.64	5.47	7.95	13.73
$\varphi (100\;years,t_{0})$	2.31	2.27	2.23	2.16	2.10	2.05	2.01	1.92	1.63	1.35	0.96

**Table 2:** Comparative summary of results in critical sections from different computational simulations.

Construction Cycle Length of One Segment	7 Days		10 Days	
Computational Simulation	W1	W2	W3	W4
Concrete Creep Model	Acc. to EN	FZ	Acc. to EN	FZ
Increase in support moment due to creep after 100 years [kNm]	−2964	−1361	−2433	−12,555
Increase in midspan moment due to creep after 100 years [kNm]	19,832	22,415	18,071	30,645
Maximum tensile stress in bottom fibers under characteristic SLS loads [MPa]	0.3	1.2	0.2	1.5
Displacements at midspan from long-term loads immediately after completion [mm]	190	261	183	224
Displacements at midspan from long-term loads, including creep, after 100 years of service [mm]	199	271	189	210

## Data Availability

The original contributions presented in this study are included in the article. Further inquiries can be directed to the corresponding author.

## Abbreviations

The following abbreviations are used in this manuscript:

AAEM	Age-Adjusted Effective Modulus method
CSM	Construction Stage Manager
FEM	Finite Element Method
FVK	Fractional Kelvin–Voigt model
FZ	Fractional Zener model
MSSB	Modified Schofield-Scott-Blair model
